# QuIN: A Web Server for Querying and Visualizing Chromatin Interaction Networks

**DOI:** 10.1371/journal.pcbi.1004809

**Published:** 2016-06-23

**Authors:** Asa Thibodeau, Eladio J. Márquez, Oscar Luo, Yijun Ruan, Francesca Menghi, Dong-Guk Shin, Michael L. Stitzel, Paola Vera-Licona, Duygu Ucar

**Affiliations:** 1 Department of Computer Science, University of Connecticut, Storrs, Connecticut, United States of America; 2 The Jackson Laboratory for Genomic Medicine, Farmington, Connecticut, United States of America; 3 Institute of Systems Genomics, University of Connecticut Health Center, Farmington, Connecticut, United States of America; 4 Center for Quantitative Medicine, Department of Cell Biology, University of Connecticut Health Center, Farmington, Connecticut, United States of America; University of Canterbury, NEW ZEALAND

## Abstract

Recent studies of the human genome have indicated that regulatory elements (e.g. promoters and enhancers) at distal genomic locations can interact with each other via chromatin folding and affect gene expression levels. Genomic technologies for mapping interactions between DNA regions, e.g., ChIA-PET and HiC, can generate genome-wide maps of interactions between regulatory elements. These interaction datasets are important resources to infer distal gene targets of non-coding regulatory elements and to facilitate prioritization of critical loci for important cellular functions. With the increasing diversity and complexity of genomic information and public ontologies, making sense of these datasets demands integrative and easy-to-use software tools. Moreover, network representation of chromatin interaction maps enables effective data visualization, integration, and mining. Currently, there is no software that can take full advantage of network theory approaches for the analysis of chromatin interaction datasets. To fill this gap, we developed a web-based application, QuIN, which enables: 1) building and visualizing chromatin interaction networks, 2) annotating networks with user-provided private and publicly available functional genomics and interaction datasets, 3) querying network components based on gene name or chromosome location, and 4) utilizing network based measures to identify and prioritize critical regulatory targets and their direct and indirect interactions. **AVAILABILITY:** QuIN’s web server is available at http://quin.jax.org QuIN is developed in Java and JavaScript, utilizing an Apache Tomcat web server and MySQL database and the source code is available under the GPLV3 license available on GitHub: https://github.com/UcarLab/QuIN/.

This is a *PLOS Computational Biology* Software paper.

## Introduction

Chromatin structure plays a major role in basic cellular functions. Advances in genomic technologies have revealed information regarding three-dimensional (3D) chromatin conformation and have shown that many regulatory elements that are distal on the linear genome map are actually in close physical proximity with each other as a result of the 3D chromatin structure. Current technologies for capturing this 3D structure include Chromosome Conformation Capture based methods (3C) [1], 4C [2], 5C [3], Hi-C [4] and Chromatin Interaction Analysis by Paired-End Tag Sequencing (ChIA-PET) [5]. These technologies identify chromatin interactions between promoters, enhancers, and other regulatory elements. The data generated by these technologies are the starting point from which we can infer distal regulatory interactions and their system-level effects by modeling them in the form of interaction networks. Moreover, integrating interaction datasets with additional data types and public repositories facilitate the discovery of regulatory elements and interactions that are critical for cellular functions and for disease biology, such as gene targets of non-coding regulatory elements harboring disease-causing Single Nucleotide Polymorphisms (SNPs). It is therefore imperative to have an easy-to-use software platform that enables biologists to model and study their chromatin interaction datasets under the light of other data sources, such as SNP databases and epigenetic marks.

Chromatin interaction data are typically visualized using a genome browser in a linear fashion, providing one-dimensional representation of the data (see S1 Fig for an example). A commonly used tool for this is the UCSC Genome Browser [6]. However, two and three dimensional representations of chromatin interactions in the form of networks and three-dimensional models can provide a global view of the interactions and facilitate the use of established network analysis methods and measures on these datasets [7]. For example, network representation of RNA Pol2 ChIA-PET data revealed that loci harboring disease-associated SNPs are differently connected in chromatin interaction networks [8]. Available tools for analyzing interaction data currently are unable to take advantage of a network representation; these tools include i) HOMER’s HiC analysis suite [9], which enables filtering and calling interactions, as well as testing for the significance of the frequency of interactions between annotations using SIMA [10], ii) HiBrowse [11], which integrates regions of interest with 3D co-localized sites; iii) GWAS3D [12], which integrates user uploaded SNPs with interaction data from ENCODE [13] to identify loci interacting with these SNPs; and iv) GenomicInteractions [14], an R package, which allows users to annotate anchors of interactions and produce summaries of the interaction data. An existing tool for building and analyzing chromatin interaction networks is CytoHiC [15], a Cytoscape [16] plugin, tailored for HiC data. However, CytoHiC is limited in annotation and query capabilities and does not enable integrating interactions with other databases through the plugin itself.

**Fig 1:**
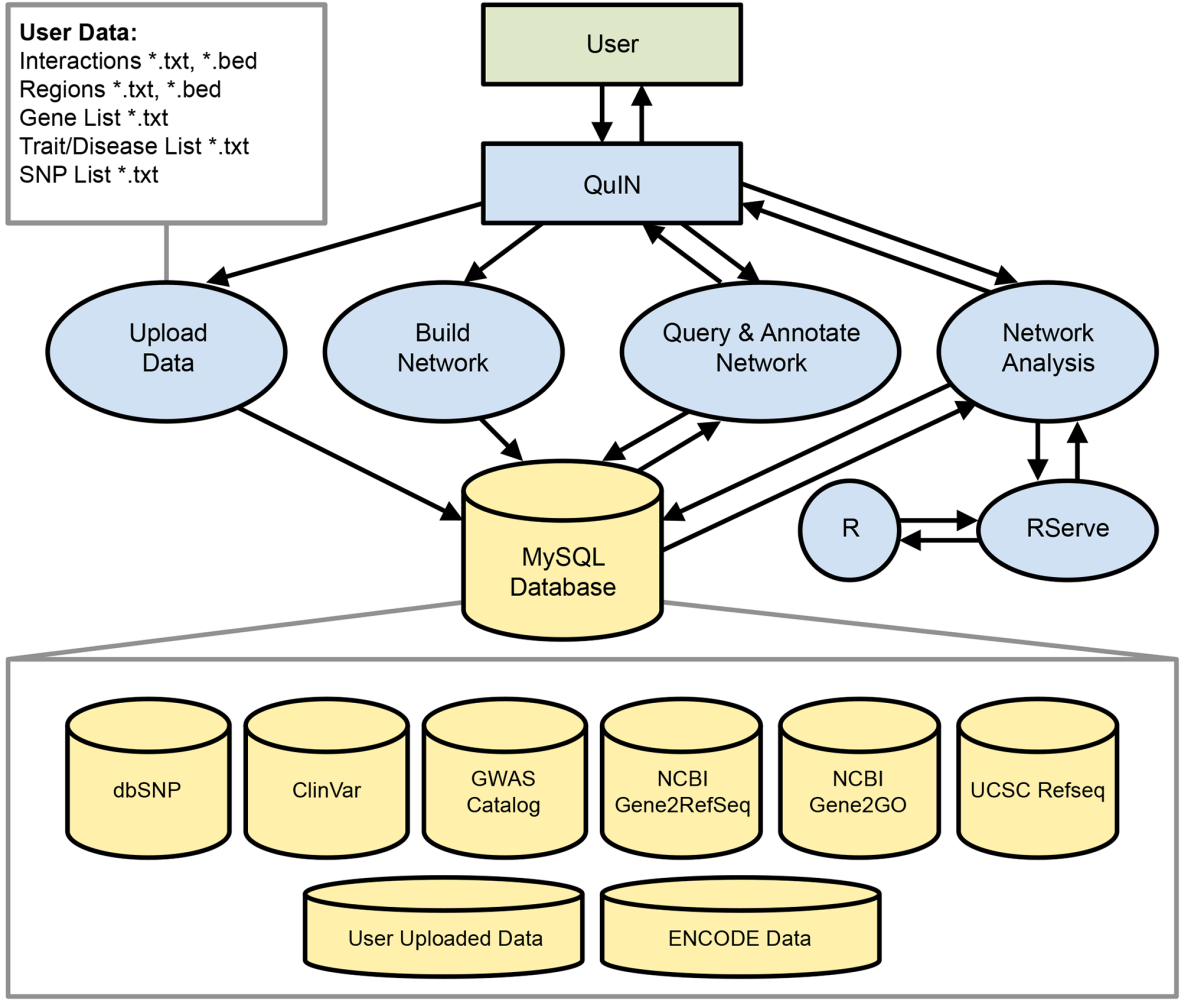
Data flow diagram of QuIN. QuIN allows users to upload diverse data types and formats and it enables building, querying, annotating, and analyzing chromatin interaction networks. QuIN also integrates publically available databases for network annotation and enrichment.

To the best of our knowledge, there is no publicly available software for the analysis of chromatin interaction networks that is web-accessible and easy to use, making it suitable for molecular biologists with no programming experience to use. To overcome the limitations of current tools, we developed a single platform for **Qu**erying and visualizing Chromatin **I**nteraction **N**etworks (QuIN) (http://quin.jax.org) (Fig 1). QuIN enables: 1) building and visualizing chromatin interaction networks from ChIA-PET or HiC interactions; 2) annotating these networks with functional information from epigenetic datasets, SNPs, gene definitions, Gene Ontology terms, other interaction networks etc.; 3) querying network components for specific genes, loci, or disease-causing SNPs; and 4) utilizing network-based algorithms and measures to prioritize genomic sites for functional validation. QuIN mines chromatin interaction datasets such as those generated by the ENCODE consortium [13] (or user supplied ones) and integrates these datasets with other functional information such as chromatin states that can be inferred from histone modification datasets or SNP databases. We summarize the features of existing tools and QuIN in S2 Fig. In summary, QuIN is designed to enable biologists to easily represent and annotate their chromatin-interaction datasets in the form of networks and to use these datasets for discovering important interactions or targets.

## Design and Implementation

### Software and Databases

QuIN is implemented in Java and JavaScript, with a MySQL database backend for storing user supplied data and local instances of the publicly available data including ClinVar [17], dbSNP [18], GWAS Catalog [19], NCBI’s Gene2Refseq and Gene2GO databases[20] and UCSC’s hg19 RefSeq database [21]. The graphical user interface is developed using Cytoscape JS (http://js.cytoscape.org/) for network visualization and JQuery/JQuery UI (https://jquery.com/) for other user interface elements. For network and other analyses, the web server communicates with R using RServe (https://rforge.net/Rserve/) to utilize existing R packages including topGO, vioplot and pheatmap to generate GO analyses, violin plots and heatmaps. Data are uploaded anonymously and privately, utilizing cookies to store the necessary information for linking the user’s browser with their uploaded data. Fig 1 summarizes the data flow and architecture of QuIN.

### Network Construction from Interaction Data

QuIN implements two approaches for constructing networks from chromatin interactions obtained via pre-processing tools such as ChIA-PET tool [22] or HOMER [9]. The first approach is based on using the interaction data alone to define network nodes by merging overlapping interaction anchors, whereas the second approach incorporates additional data, such as open chromatin sites from DNASE-Seq [23] or ATAC-Seq [24] technologies, to define or refine the nodes in the network, which is useful to filter out potential false positive interactions. Once nodes are defined, pairs of nodes are connected by identifying chromatin interactions whose anchors overlap the boundaries of these nodes; these interactions thus define the edges of the network. QuIN keeps track of the number of interactions that connect node pairs as edge weights. Finally, a breadth first search method is carried out to isolate the connected components of the network. A more detailed description of the algorithms implemented for network construction can be found in S1 Text.

### Annotating and Analyzing Networks

**Fig 2:**
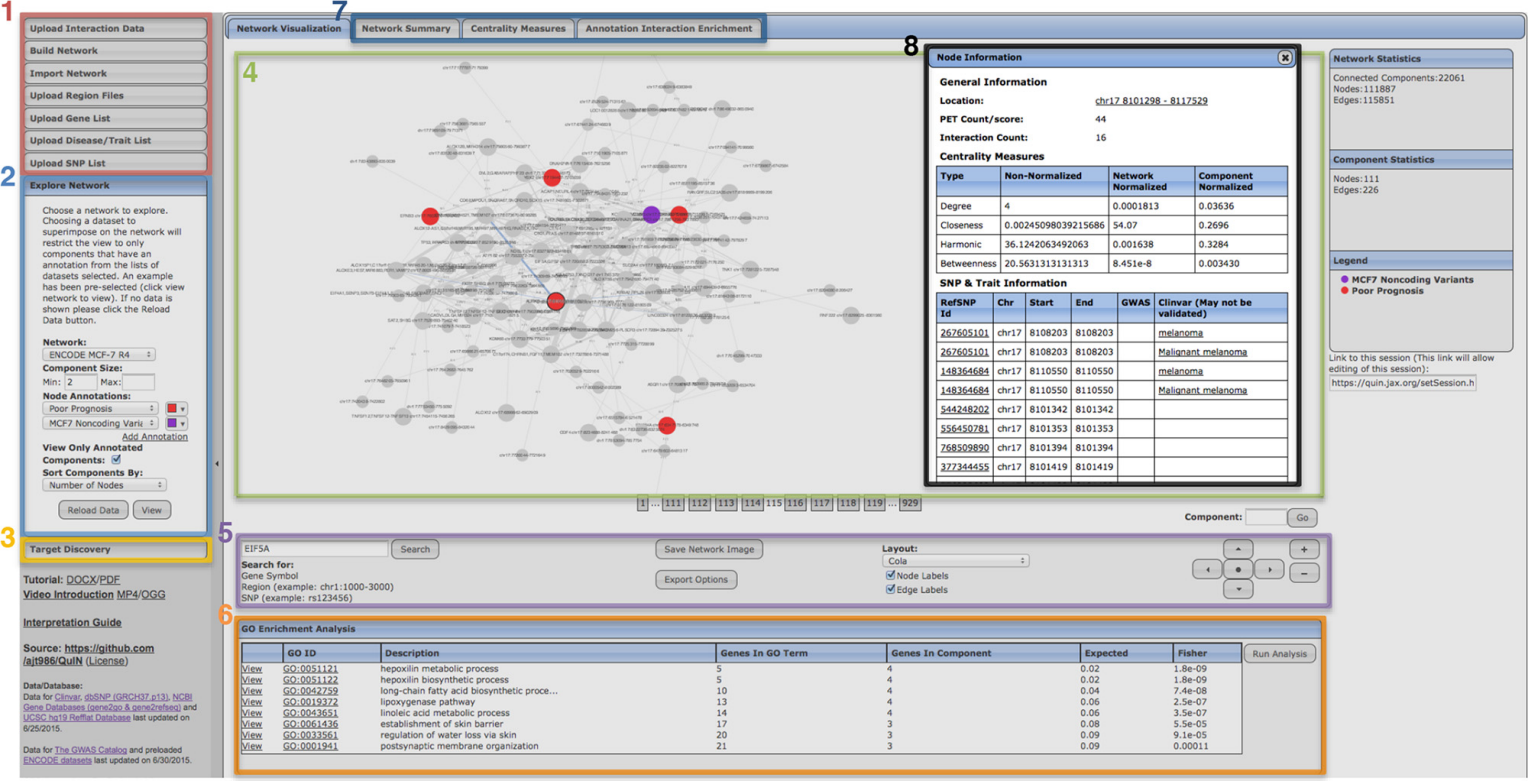
A screenshot of QuIN’s web interface highlighting its features. (1) menus for uploading data and building networks, (2) options for visualizing and annotating a network, (3) target discovery menu for visualizing and exporting direct and indirect targets from source annotations to target annotations (4) network visualization panel, (5) options for searching, querying, or exporting the network, (6) the menu for performing GO Enrichment Analysis on the current subnetwork, (7) tools for summarizing network construction statistics, centrality measures and enrichment of interactions between annotations, (8) dialog box showing additional information about a selected node, including centrality measures, SNPs, and associated diseases.

Users can annotate interaction networks by uploading various types of datasets including genomic regions, genes, SNPs, and diseases/traits listed in the GWAS catalog [19]. Once annotated, QuIN offers a diverse set of measures and algorithms for further analyses of the network. Previous studies have shown that network characteristics of chromatin interactions can be linked to regulatory or three-dimensional structural properties of genes and gene products [8,25]. For example, we have shown that hubs (nodes with high degree) and spokes (nodes with low degree) exhibit distinct functional and etiological properties [8]. Among these, we noted that the hubs lacked disease-associated SNPs, suggesting an evolutionary selection that favors the nonrandom spatial clustering of the genomic domains. Currently, implemented network measures within QuIN include: (1) connectivity degree which measures the number of edges connected to a node, quantifying the number of interactions to one particular loci; (2) betweenness, measuring the number of times a node exists on the shortest path between all other pairs of nodes, suggesting loci which may be essential for others sites to interact; and (3) closeness & harmonic centrality, which measure the average shortest paths to all other nodes in the component or network, suggesting loci which are more central and are interacting with different locus. To further understand the connectivity between annotations and to utilize network topology for biological discovery, QuIN also provides a target discovery function, which allows users to export all of the shortest paths from source nodes to target nodes, collecting all direct and indirect interactions between two sets of annotated nodes. Additionally, QuIN provides methods for analyzing the frequency of interactions between pairs of annotations to reveal whether interactions between nodes annotated with certain features (e.g. nodes harboring SNPs vs. nodes overlapping with enhancers) are enriched relative to the random expectation. QuIN provides two options for calculating expected frequencies between two annotations: (1) by permutation to randomize the network annotations, and (2) by calculating a theoretical expected frequency. Significance of an observed frequency is calculated using (1) one-tailed binomial test p-values and (2) p-values with respect to the null distribution derived from permutations. Further details on statistical tests are provided in S1 Text. Finally, to enable using other existing methods for network analyses, the chromatin interaction network can be exported in standard Graph Modelling Language (.gml) format, allowing the network generated by QuIN to be imported into existing tools that support this format, such as Cytoscape [16]. A screenshot of the web based graphical user interface summarizing QuIN’s features is shown in Fig 2.

## Results

### A case study to discover gene targets of non-coding variants in breast cancer cell line MCF-7

**Fig 3:**
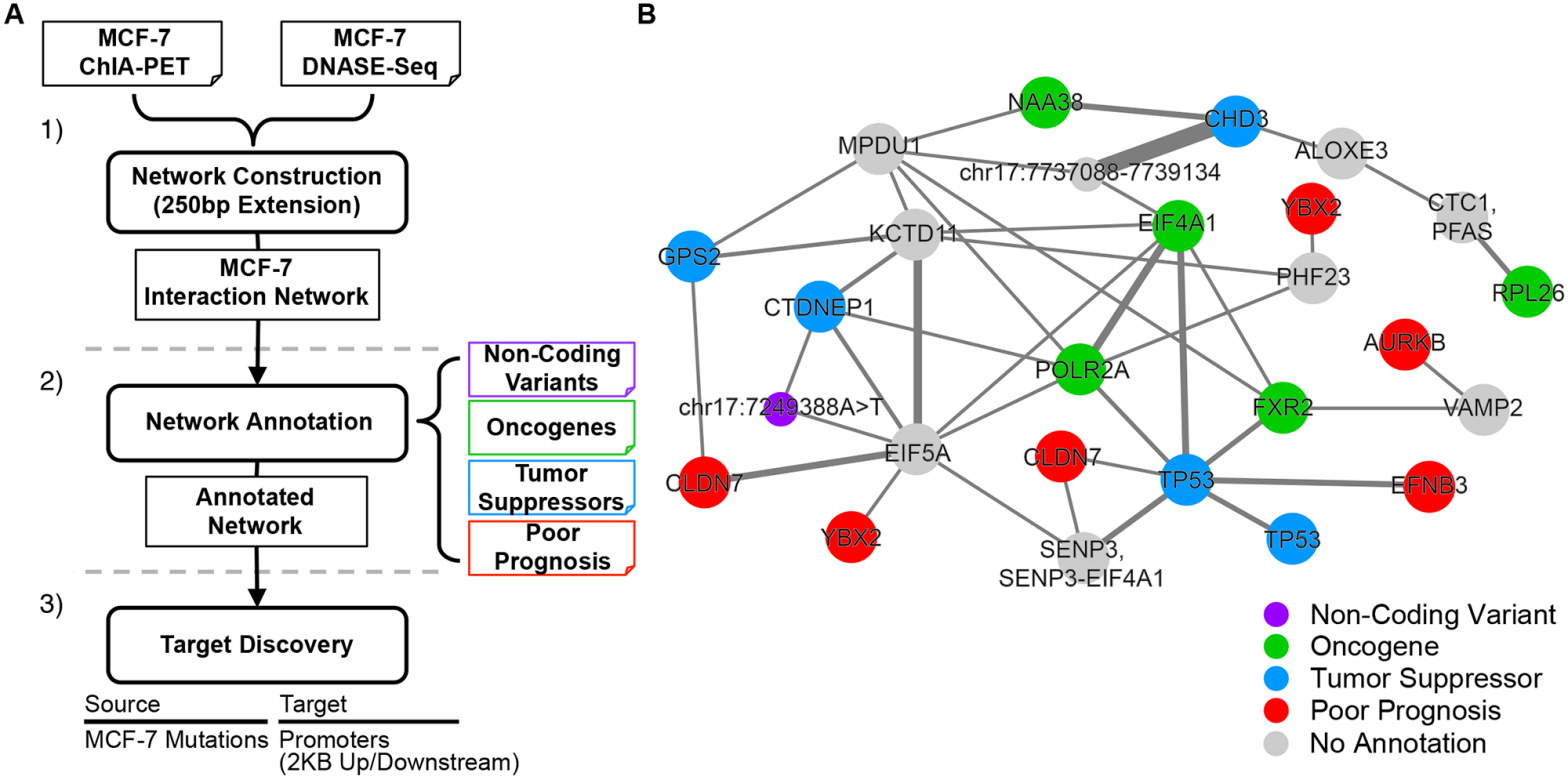
A breast cancer case study with QuIN. (A) Workflow of the case study analysis. (1) Upload the DNASE-Seq and Interaction data into QuIN, constructing an MCF-7 interaction network where each node represents an open chromatin site. (2) Annotate the network with Non-Coding Variants (NCVs) in MCF-7 and cancer associated gene lists. (3) Perform target discovery between NCVs (source) and promoters and cancer gene lists (targets) and find all direct and indirect associations between NCVs and their gene targets. (B) A simplified network example showing the interactions between a node harboring an NCV (shown in purple) and known oncogenes (green), genes associated with poor prognosis in breast cancer (red), and tumor suppressor genes (blue). Nodes shown were selected based on their overlap with an annotation or if the node is necessary to connect the NCV to the annotated node. Width of the edges correspond to the relative number of paired end tags supporting the edge.

We demonstrate the core functionalities of QuIN by conducting a case study using RNA Pol2 ChIA-PET data in the breast cancer cell line MCF-7. For this analysis, we constructed an interaction network in MCF-7 using ChIA-PET (GSM970209) and DNASE-Seq (GSM816627) data generated by the ENCODE consortium [13]. This resulted in an MCF-7 network comprising 59,082 nodes, 65,308 edges, and 8,133 connected components. In the absence of chromatin interaction datasets, variants are associated to the closest gene transcription start site (TSS). However, increasing evidence points out to the importance of noncoding variants in disease biology [26] and the frequency of distal interactions between genes and their non-coding regulatory elements. Hence, we sought to use the MCF-7 interaction network to discover gene targets of non-coding variants that are likely to be associated with breast cancer. QuIN can be employed to conduct similar analyses using coding variants. However, coding variants have a clearer phenotypic outcome: amino-acid change in the corresponding gene sequence and a possible effect on that protein’s function or stability. Therefore, the primary impact of a coding variant is expected to be local and just restricted to the gene (and the protein) harboring the variant. Workflow of our case study is explained in Fig 3A with an example annotated network shown in Fig 3B.

**Fig 4:**
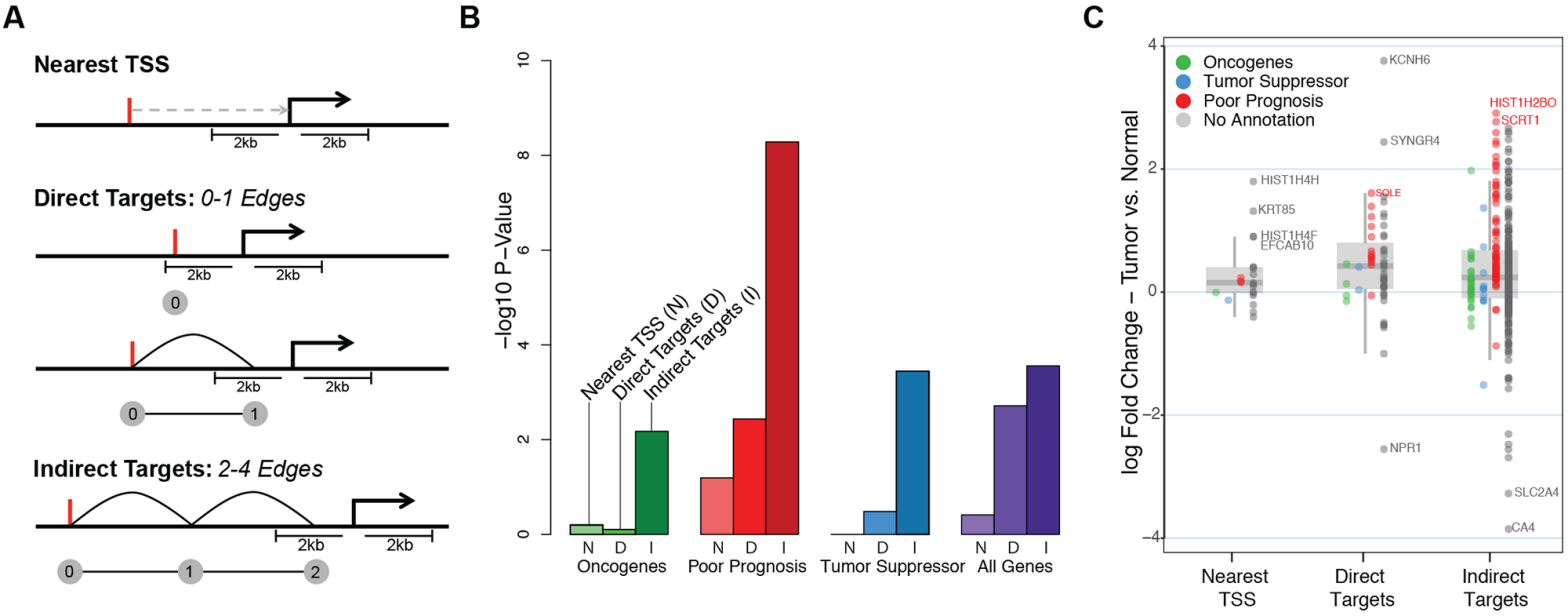
Comparison of ChIA-PET gene targets with nearest gene targets. (A) A cartoon describing different approaches to associate NCVs with gene targets (Nearest TSS, Direct Targets, & Indirect Targets). (B) Enrichment p-values (based on Fisher’s exact test) of cancer related genes (known oncogenes (green), tumor suppressor genes (blue), poor prognosis genes (red), and the combined gene list (purple)) among NCV gene targets obtained via nearest TSS, direct target, indirect target associations. (C) Boxplot showing the differential expression (between cancer and normal tissues) for NCV target genes obtained via nearest TSS, direct target, indirect target associations.

For this purpose, we first annotated the MCF-7 interaction network with non-coding variants (NCV) found in the MCF-7 cell line obtained from the COSMIC database [27] (cancer.sanger.ac.uk), which catalogues somatic mutations in cancer, and identified 36 NCVs that overlap with nodes in the network. To identify distal and proximal gene targets of these potentially disease-causing variants in the MCF-7 network, we used the target discovery feature of QuIN and identified all shortest paths from nodes harboring NCVs to nodes associated with promoters (defined as 2kb up/downstream of TSS using RefSeq gene definitions). We identified the ‘direct targets’ of these NCVs by finding the genes that are either within the same node as the variant or one edge away in the network. We also captured ‘indirect targets’ by further expanding the search and finding genes that are 2–4 edges away from the NCV containing node, which we determined by studying the enrichment of cancer-related genes among targets identified via different edge distances (S3 Fig) (see S1 Text for details and Fig 4A for definitions). Recent studies have shown that indirect interactions are useful for predicting gene co-expression patterns [28] and identifying complex regulatory interactions such as enhancer-enhancer-promoter interactions [29], pointing out to the significance of identifying and studying indirect chromatin interactions through a network-based approach. In summary, our analyses revealed 90 genes that are ‘direct targets’ and 638 genes that are ‘indirect targets’ of the NCVs in the MCF-7 network. (S1 Table).

To understand whether gene targets obtained from ChIA-PET interaction networks can be useful in discovering genes with potential implications in breast cancer, we compared the gene lists obtained from MCF-7 interaction networks (both direct and indirect targets) with the gene list obtained via ‘nearest TSS’ annotation (Fig 4A). Using the nearest TSS approach, we identified 30 genes, which we compared against direct (90) and indirect targets (638) obtained from the ChIA-PET network analyses. First, we annotated our MCF-7 network with cancer associated genes including known oncogenes [30,31,32], tumor suppressor genes identified by [33], genes previously associated with poor prognosis in breast cancer patients [34], and a unified list of cancer related genes including all previous lists and other gene lists [27,35,36] (further details of these lists are provided in S1 Text).

Next we calculated the enrichment of known breast cancer-related genes in targets discovered by i) nearest TSS; ii) direct ChIA-PET targets; and iii) indirect ChIA-PET targets using Fisher’s exact test. Our analyses showed that ChIA-PET based targets are significantly more enriched for cancer-related genes than nearest gene targets (Fig 4B). For example, for genes associated with poor prognosis in breast cancer, we have shown that the enrichment Fisher’s p-value for direct targets is 3.65 × 10^−3^, indirect targets is 5.18 × 10^−9^, whereas nearest gene targets is 6.39 × 10^−2^ (Fig 4B and S4 Fig, S2 Table). This analysis also revealed that with the help of chromatin interaction networks and our tool QuIN, we can capture gene targets of NCVs that are more likely to be relevant for the disease than nearest TSS assignments, even if they are separated via multiple edges in the network. To further assess their relevance to breast cancer, we studied these three lists of genes by comparing their expression levels among TCGA samples [37]. For this, we first calculated the differential expression of genes between breast cancer samples and normal breast epithelium samples using TCGA RNA-seq data. We found that targets discovered by ChIA-PET include genes that are more differentially expressed between cancer and normal tissues in comparison to nearest gene targets (Fig 4C). Moreover, we have also observed that even indirect targets of the NCVs could be disease relevant, which highlights the system-level impact of disease-causing variants and the importance of studying these interactions at the network level. Similarly, we observed that gene targets discovered using ChIA-PET tend to show more differential expression between the MCF-7 line and MCF-10A, a non-cancer mammary epithelial cell line, using both single-cell and bulk RNA-seq datasets [38] (GSE52712) (S5 Fig). As an example, Fig 3B and S6 Fig show a simplified subnetwork revealing the indirect relationships of a region harboring an NCV to genes associated with cancer, including the well-known tumor repressor *TP53* and multiple breast cancer-associated genes, such as *EIF4A* [39], *EIF5A* [40,41], *AURKB* [42] and *CLDN7* [43]. Furthermore, we show linear and network representations of the chromatin interactions in an example locus along with the gene expression fold changes for selected genes (S1 Fig using TCGA and S7 Fig using MCF-7 and MCF-10A gene expression datasets). Our analyses revealed the potential impact of NCVs on cancer-related gene regulatory programs through their chromatin connectivity.

To further interrogate the regulatory implications of ChIA-PET interactions, we calculated co-expression of gene pairs using 914 breast cancer samples from the TCGA data. Our results showed that direct interactions overall have the highest co-expression values. Yet, indirect interactions have significantly higher co-expression levels when compared to non-interacting gene pairs, implying the co-regulation of genes connected via direct or indirect chromatin interactions (S8 Fig). In addition, we integrated MCF-7 network with protein-protein interactions obtained from the STRING database [44]. After super-imposing the protein-protein interactions onto the MCF-7 network using QuIN, we identified genes exhibiting indirect chromatin interactions that also have direct protein-protein interactions (S9 Fig). One such example is TP53 and AURKB, which show a direct protein-protein interaction while indirectly interacting in the chromatin interaction network. This further increases our evidence of the importance of indirect interactions when analyzing chromatin interaction data, which could be relevant in multiple biological contexts.

This case study demonstrates how researchers can use QuIN to easily interrogate chromatin interaction datasets in conjunction with other data sources, such as variant catalogs, to identify and prioritize candidate regulatory elements relevant for normal and abnornal cell function. It also illustrates the power of network-based approaches to facilitate data-driven selection of gene targets of disease-associated mutations and polymorphisms, in comparison to the traditional selection of gene targets based on linear genomic distance, which does not take into consideration the three-dimensional nature of regulatory interactions in the genome.

## Availability and Future Directions

To the best of our knowledge, QuIN is the first web accessible and easy to use platform for analyzing 3D chromatin interaction datasets and we plan to extend its capabilities in multiple areas. First, we plan to implement algorithms for mining network motifs that might be important for biological functions, taking further advantage of network theory approaches. Second, we intend to provide more genomes beyond hg19 to support a broader range of species and experiments. Future extensions include expanding QuIN’s data integration capabilities with the addition of expression data, including their visualization in the context of the chromatin interaction network. Additionally, as chromatin interaction datasets become abundant, it will be important to implement features for comparing networks in terms of interaction losses and gains, which will allow users to infer potential regulatory changes resulting from these differences.

QuIN is an open source project released under the GNU General Public License Version 3 and is available on GitHub (https://github.com/UcarLab/QuIN/) and in S1 software. In addition, we have an online web server (http://quin.jax.org) providing a publicly available working version of our software.

## Supporting Information

S1 Fig An example loci using linear and three-dimensional representation with TCGA gene expression data. (A) BASIC browser screenshot of the region corresponding to the network example in Fig 3B and S6 Fig. Genes shown have been selected based on representation within the network. (B) Network representation of the same region with nodes aligned based on overlapping markers, genes, and interactions. (C) Tumor vs. Normal fold change of TCGA expression data for selected genes found within the network. The TSS nearest to the non-coding variant, *KCTD11*, has been highlighted in red.
(TIF)

S2 Fig Table summarizing features of six computational tools developed to analyze chromatin interaction datasets. Of the tools reviewed, only QuIN, CytoHiC and GenomicInteractions have provided methods for interpreting chromatin interactions as a network, with GenomicInteractions only providing the ability to construct a network by using other R packages. Considering the accessibility of the tools, QuIN, HiBrowse, and GWAS3D have been developed as web-based applications, allowing immediate access to the tools through web browsers while eliminating the steps of installing and setting up the software before use. Though a majority of the tools support the ability for users to analyze their own interaction datasets, GWAS3D alternatively only allows users to upload SNPs of interest to analyze with chromatin interaction data available with the tool. All of the tools have shown some integration with public data/databases with varying levels of comprehensiveness, however CytoHiC requires other Cytoscape plugins to achieve this functionality and HOMER’s Hi-C suite fulfills this by being part of a larger package, providing similar integration by pipelining the data with other HOMER command-line tools. For analyzing the frequency of interactions between annotations, only QuIN, HiBrowse, and HOMER’s Hi-C suite provide methods for evaluating the significance of these frequencies. Finally, QuIN proves to be the only tool that offers the ability to systematically discover both direct and indirect targets, taking advantage of the network representation to determine indirect targets of a node of interest.
(TIF)

S3 Fig P-Values of enrichment of cancer gene lists in NCV target genes (Fisher’s exact test) at various indirect edge cutoffs. (TIF)

S4 Fig Enrichment analyses of gene targets. Enrichment p-values (based on Fisher’s exact test) of cancer-related genes (known oncogenes (green), known tumor suppressor genes (yellow), genes associated with good (orange) and poor prognosis (red), oncogenes and tumor suppressor genes identified by Davoli et al (2013) (pink and blue)), and the union of all cancer related genes (purple) in NCV gene targets obtained via nearest tss, direct targets, indirect targets, direct and indirect target methods.
(TIF)

S5 Fig Differential gene expression analyses for target genes. Boxplots showing the differential expression (between cancer and normal tissues) for NCV target genes obtained via nearest TSS, direct target, indirect target associations for (A) MCF-7 vs. MCF-10A Single Cell and (B) MCF-7 vs. MCF-10A Bulk samples.
(TIF)

S6 Fig An example network. Network image generated and saved using QuIN corresponding to Fig 3B, displaying exact position of nodes as well as all promoters overlapping the nodes. Values of edges indicate the number of paired end tags and the total number of interactions in parenthesis.
(TIF)

S7 Fig An example locus using linear and three-dimensional representation with MCF-7 and MCF-10A gene expression data. (A) BASIC browser screenshot of the region corresponding to the network example in Fig 3B and S6 Fig. Genes shown have been selected based on representation within the network. (B) Network representation of the same region with nodes aligned based on overlapping markers, genes, and interactions. (C) MCF-7 vs. MCF-10A fold change for selected genes found within the network. The nearest TSS of the non-coding variant, KCTD11, has been highlighted in red.
(TIF)

S8 Fig Direct and indirect interacting gene pairs exhibit elevated gene expression correlations. Gene expression correlations between gene pairs connected via direct and indirect interactions in the MCF-7 ChIA-PET network, compared to correlations between not interacting genes.
(TIF)

S9 Fig QuIN enables superimposing other types of interactions with chromatin interactions. An example list of interactions from the STRING database (A) superimposed on a ChIA-PET chromatin interaction subnetwork (B).
(TIF)

S1 Text Supplementary Methods. (PDF)

S1 Table Target discovery output for the case study. (CSV)

S2 Table Cancer gene hits among gene target lists. (CSV)

S1 Software Archive for QuIN’s source code. (7Z)
