# Supplementary figures and images for: QuIN: A Web Server for Querying and Visualizing Chromatin Interaction Networks
Source: PLoS Comput Biol. 2016 Jun 23;12(6):e1004809. doi: 10.1371/journal.pcbi.1004809 (PMC4919057; doi:10.1371/journal.pcbi.1004809)

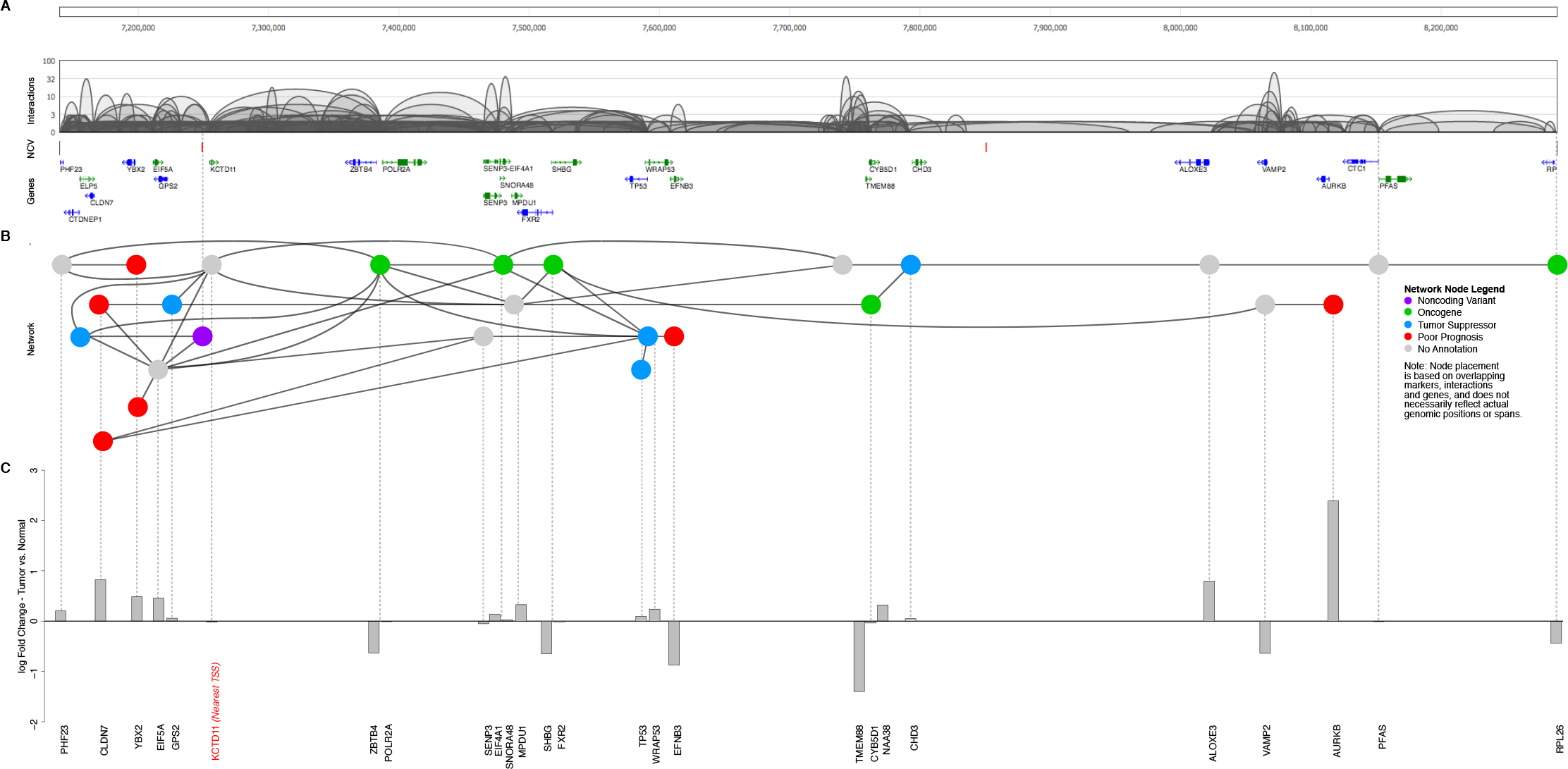

Supplement: S1 Fig — (A) BASIC browser screenshot of the region corresponding to the network example in Fig 3B and S6 Fig. Genes shown have been selected based on representation within the network. (B) Network representation of the same region with nodes aligned based on overlapping markers, genes, and interactions. (C) Tumor vs. Normal fold change of TCGA expression data for selected genes found within the network. The TSS nearest to the non-coding variant, KCTD11, has been highlighted in red. (TIF) [file pcbi.1004809.s001.tif]

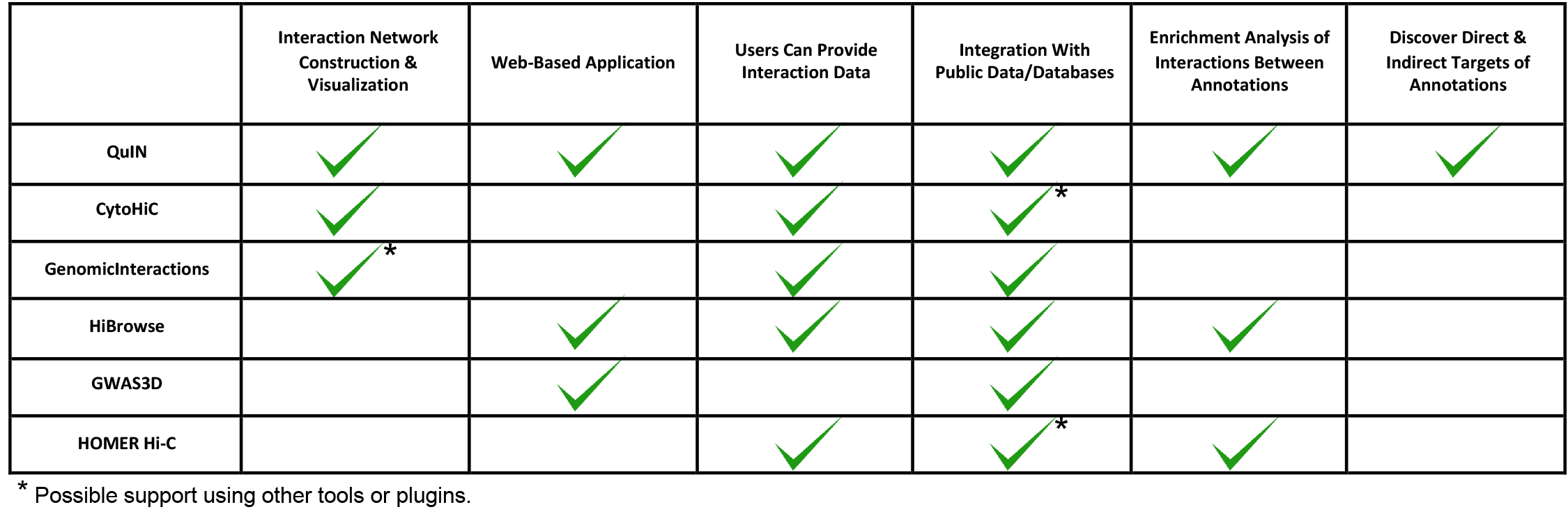

Supplement: S2 Fig — Of the tools reviewed, only QuIN, CytoHiC and GenomicInteractions have provided methods for interpreting chromatin interactions as a network, with GenomicInteractions only providing the ability to construct a network by using other R packages. Considering the accessibility of the tools, QuIN, HiBrowse, and GWAS3D have been developed as web-based applications, allowing immediate access to the tools through web browsers while eliminating the steps of installing and setting up the software before use. Though a majority of the tools support the ability for users to analyze their own interaction datasets, GWAS3D alternatively only allows users to upload SNPs of interest to analyze with chromatin interaction data available with the tool. All of the tools have shown some integration with public data/databases with varying levels of comprehensiveness, however CytoHiC requires other Cytoscape plugins to achieve this functionality and HOMER’s Hi-C suite fulfills this by being part of a larger package, providing similar integration by pipelining the data with other HOMER command-line tools. For analyzing the frequency of interactions between annotations, only QuIN, HiBrowse, and HOMER’s Hi-C suite provide methods for evaluating the significance of these frequencies. Finally, QuIN proves to be the only tool that offers the ability to systematically discover both direct and indirect targets, taking advantage of the network representation to determine indirect targets of a node of interest. (TIF) [file pcbi.1004809.s002.tif]

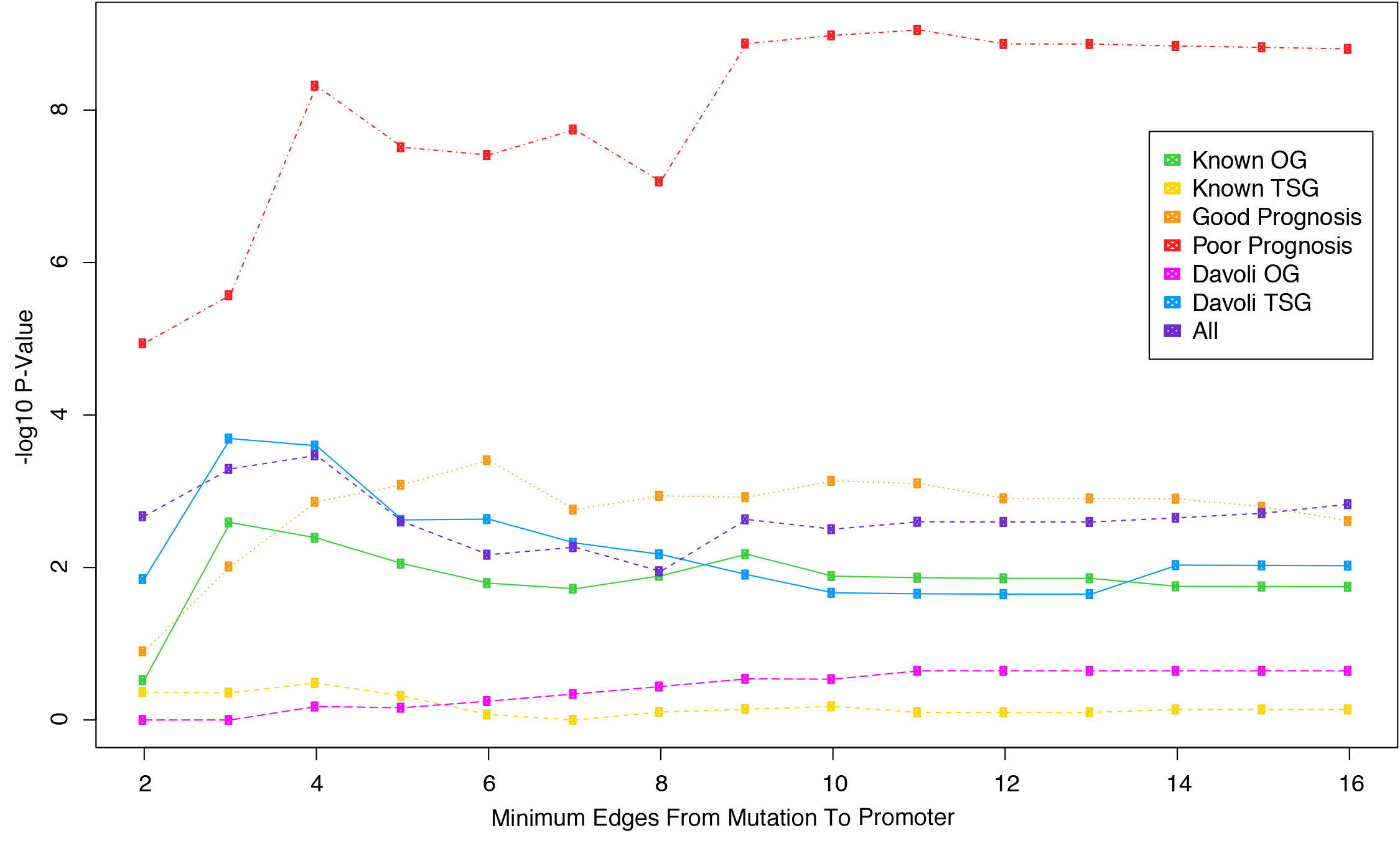

Supplement: S3 Fig — (TIF) [file pcbi.1004809.s003.tif]

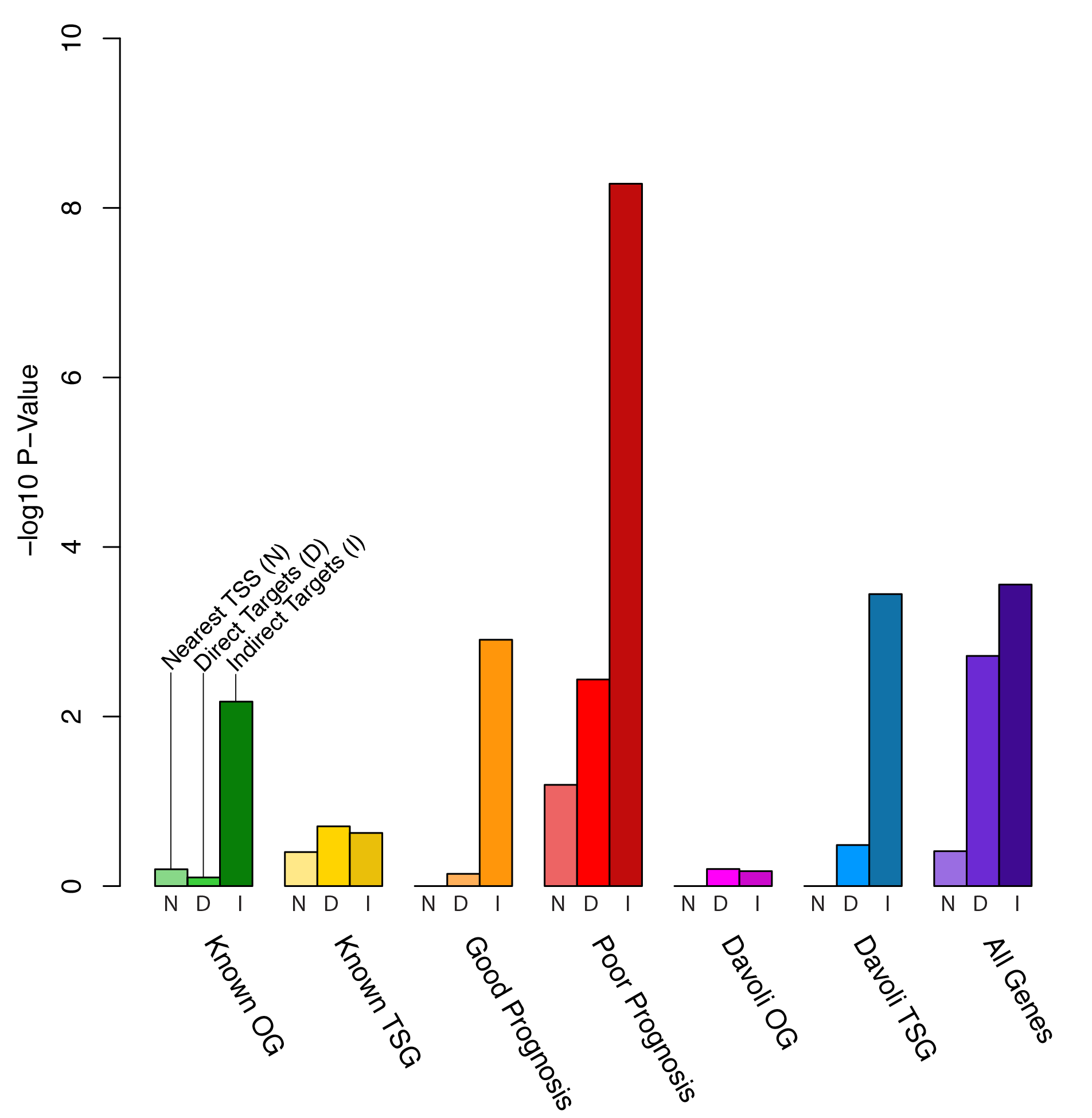

Supplement: S4 Fig — Enrichment p-values (based on Fisher’s exact test) of cancer-related genes (known oncogenes (green), known tumor suppressor genes (yellow), genes associated with good (orange) and poor prognosis (red), oncogenes and tumor suppressor genes identified by Davoli et al (2013) (pink and blue)), and the union of all cancer related genes (purple) in NCV gene targets obtained via nearest tss, direct targets, indirect targets, direct and indirect target methods. (TIF) [file pcbi.1004809.s004.tif]

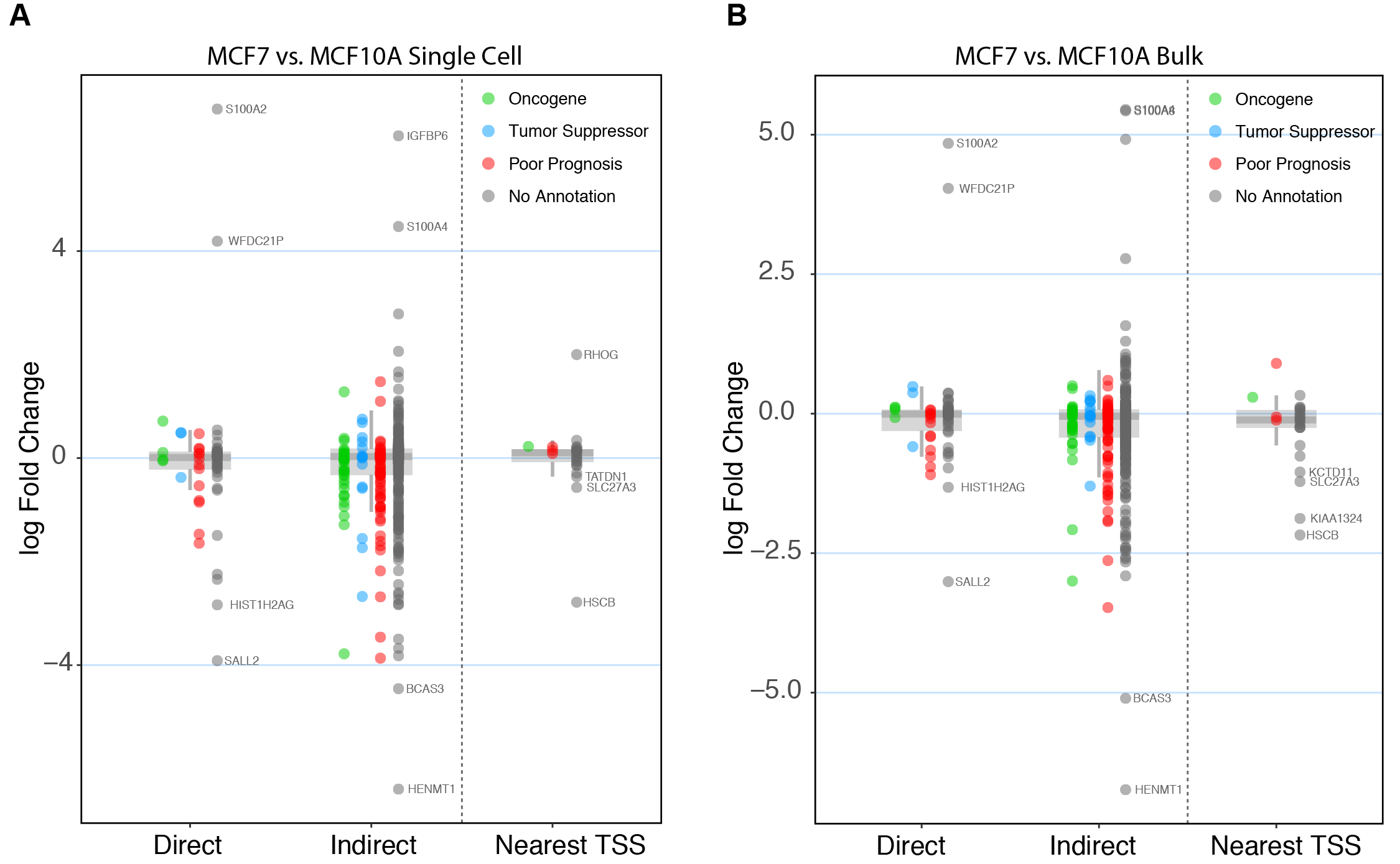

Supplement: S5 Fig — Boxplots showing the differential expression (between cancer and normal tissues) for NCV target genes obtained via nearest TSS, direct target, indirect target associations for (A) MCF-7 vs. MCF-10A Single Cell and (B) MCF-7 vs. MCF-10A Bulk samples. (TIF) [file pcbi.1004809.s005.tif]

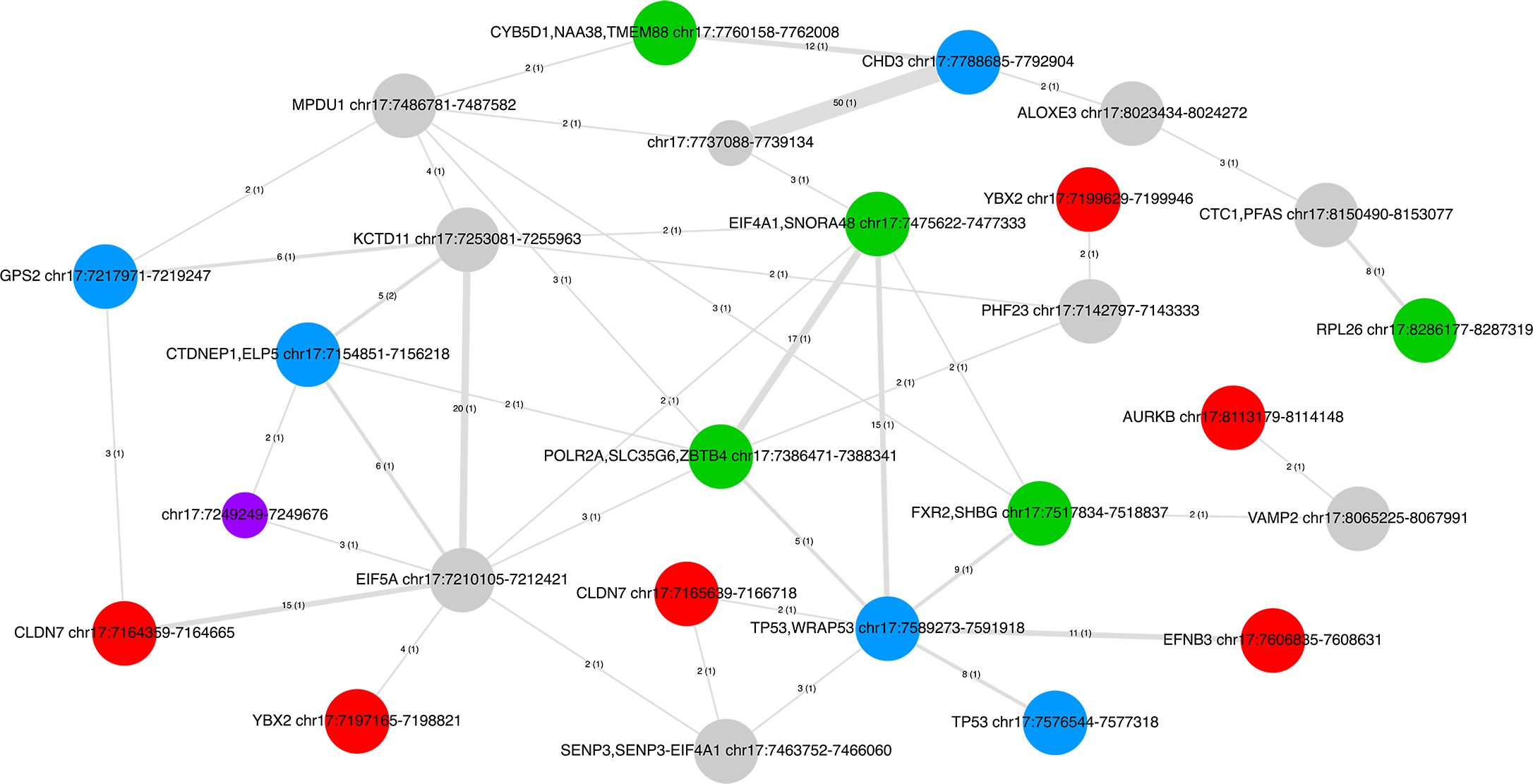

Supplement: S6 Fig — Network image generated and saved using QuIN corresponding to Fig 3B, displaying exact position of nodes as well as all promoters overlapping the nodes. Values of edges indicate the number of paired end tags and the total number of interactions in parenthesis. (TIF) [file pcbi.1004809.s006.tif]

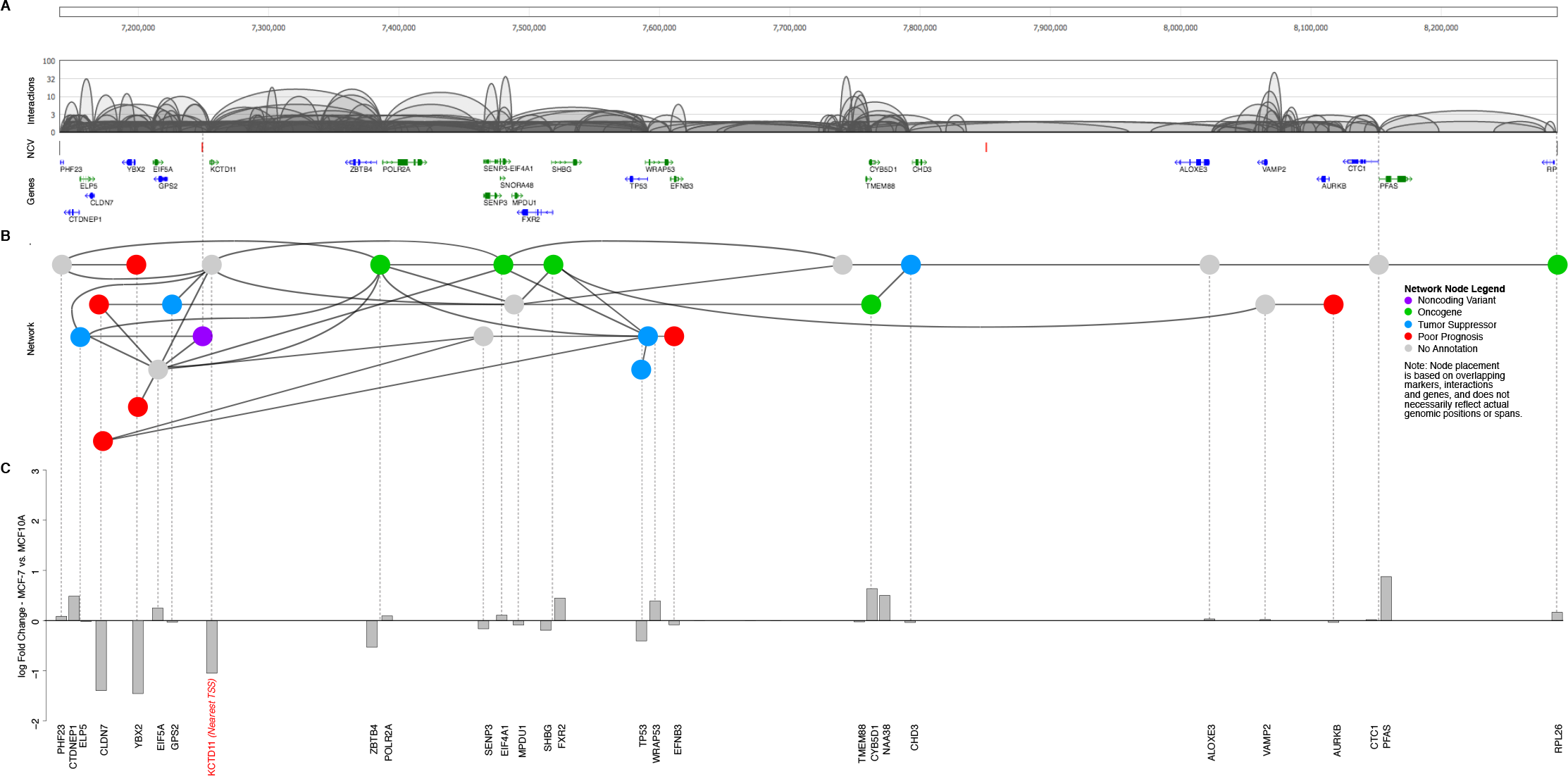

Supplement: S7 Fig — (A) BASIC browser screenshot of the region corresponding to the network example in Fig 3B and S6 Fig. Genes shown have been selected based on representation within the network. (B) Network representation of the same region with nodes aligned based on overlapping markers, genes, and interactions. (C) MCF-7 vs. MCF-10A fold change for selected genes found within the network. The nearest TSS of the non-coding variant, KCTD11, has been highlighted in red. (TIF) [file pcbi.1004809.s007.tif]

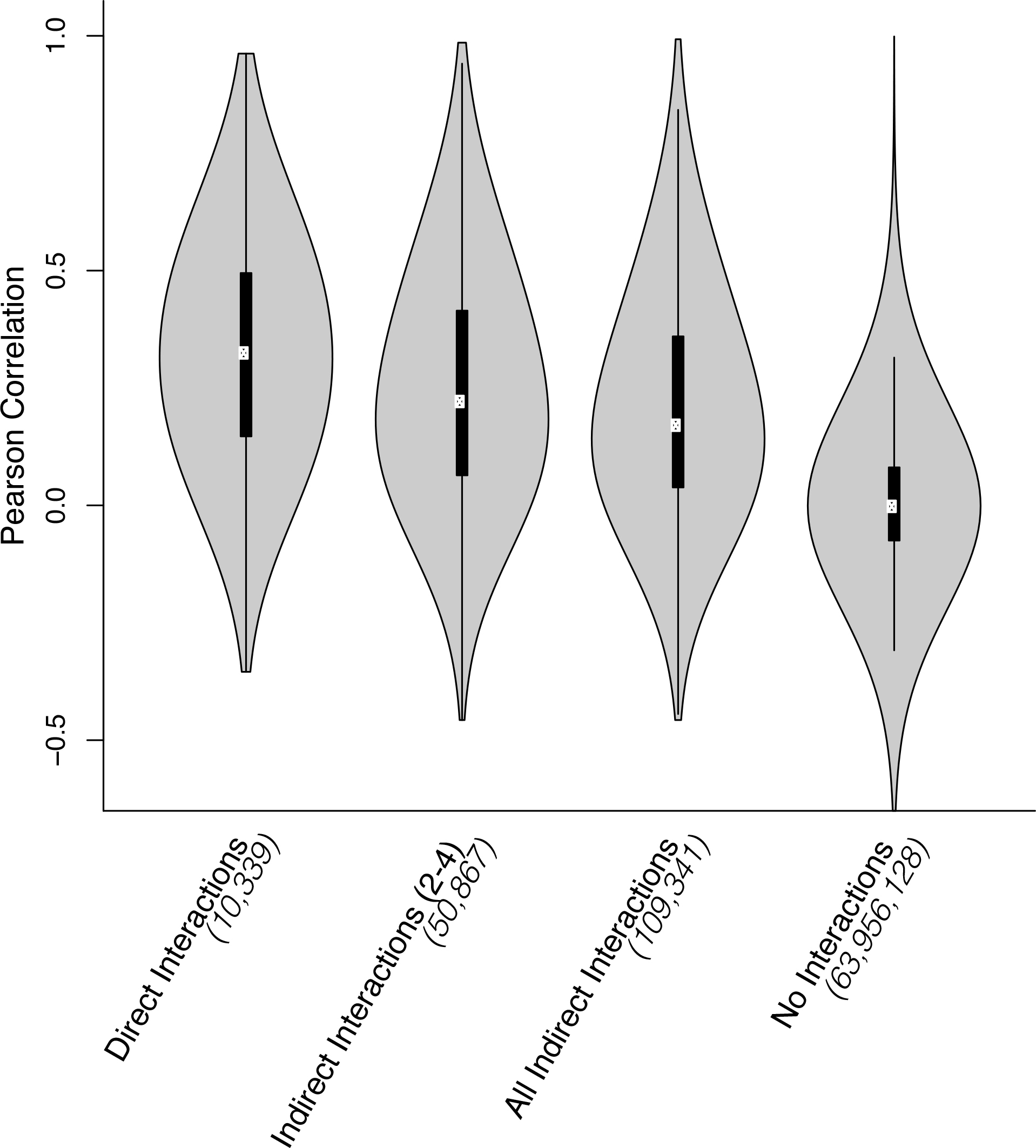

Supplement: S8 Fig — Gene expression correlations between gene pairs connected via direct and indirect interactions in the MCF-7 ChIA-PET network, compared to correlations between not interacting genes. (TIF) [file pcbi.1004809.s008.tif]

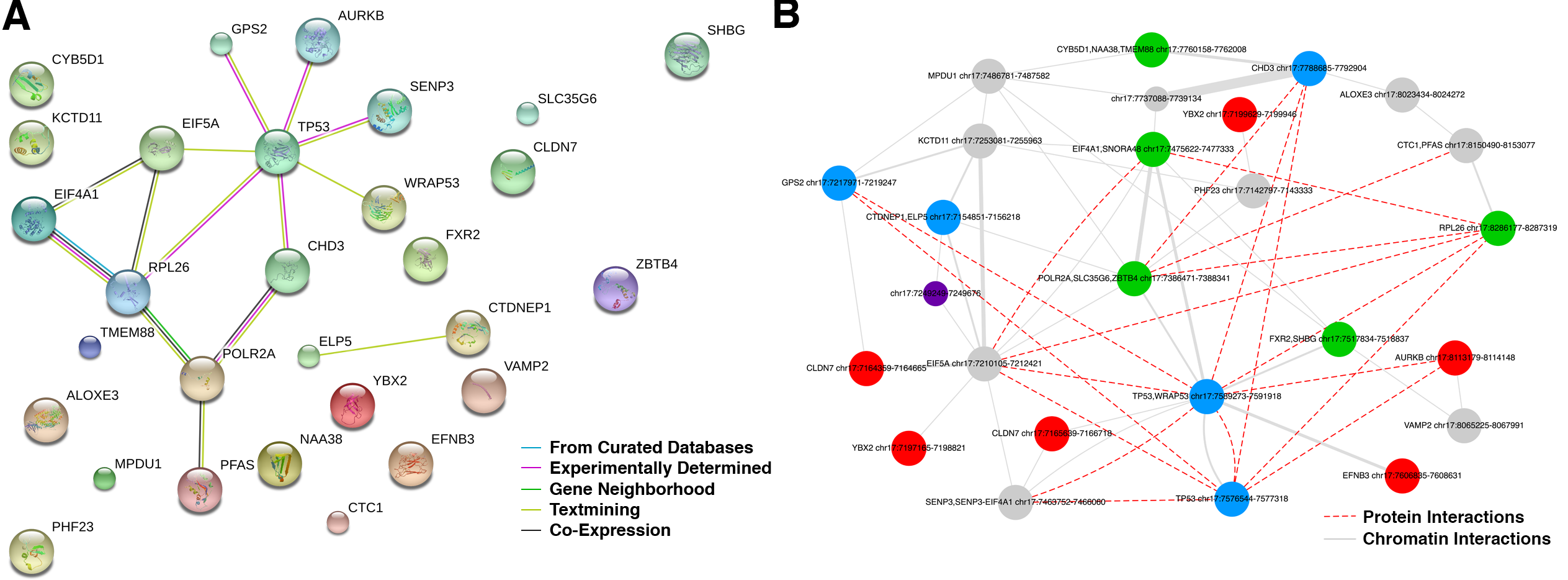

Supplement: S9 Fig — An example list of interactions from the STRING database (A) superimposed on a ChIA-PET chromatin interaction subnetwork (B). (TIF) [file pcbi.1004809.s009.tif]
