# Supplementary material for: QuIN: A Web Server for Querying and Visualizing Chromatin Interaction Networks
Source: PLoS Comput Biol. 2016 Jun 23;12(6):e1004809. doi: 10.1371/journal.pcbi.1004809 (PMC4919057; doi:10.1371/journal.pcbi.1004809)
Supplement: S1 Text — (PDF) [file pcbi.1004809.s010.pdf]

Supplementary Methods

We describe the methods used for constructing and annotating chromatin interaction networks in detail as well as methods used for analyzing these networks in QuIN.

Network Construction

Chromatin interaction networks are constructed in three steps: (1) Node Creation, (2) Edge Creation, and (3) Connected Component Discovery. We describe each method in detail below including both available methods for defining nodes within the network.

Node Creation

Method 1: Pre-Defined Node Locations

With additional data provided for defining nodes, nodes are initially created using the node definitions provided. If the regions within the data are found to be overlapping, then a step is performed to merge overlapping regions together into one region. The remainder of the algorithm focuses on determining the interaction anchors that overlap with the nodes in the network which is necessary for defining the edges of the network. For this, both nodes and anchors are separated based on chromosome where each list is sorted by start position. For each chromosome, the corresponding list of nodes and anchors are iterated concurrently as follows: Select the first node in the sorted list and iterate over all anchors until the next anchor's start position is greater than the node's end position. Each anchor is compared with the current node as well as the next node in the list to determine whether or not the anchor overlaps with either of these nodes after extending the nodes by the extend parameter in both directions. If an anchor overlaps with both nodes with extension, another comparison is made without extension. If only one node overlaps with the anchor without extension, then the anchor is assigned to the overlapping node. If both nodes are still overlapping with the anchor, then the node that is overlapping with the anchor greater than half its size is assigned. In the case that neither node overlaps without extension, then a final comparison is made with extension again, checking if the anchor overlaps with one node (with extension) greater than half the length of the

anchor while overlapping the other node less than half. If no assignment can be made, then the interaction is categorized as ambiguous and will not be considered in the edge creation step.

Method 2: Defining Nodes with Interaction Anchors

Using only the interaction data, the second method defines nodes by merging interaction anchors. To merge, anchors are first separated based on chromosome and sorted by start position. For each chromosome, the corresponding list is iterated over once, initializing a list to maintain anchors to merge and maintaining the greatest end position seen. If the next anchor in the iteration has a start position less than that of the greatest end position seen then the anchor is added to the current list and the end position is updated if the new anchor's end position is greater than the current greatest end position. If the next anchor's start position is greater than the current greatest end position, then the current list of anchors defines a new node in the network and a new list is created to begin determining the next node in the network. Performing this procedure over all chromosome defines all nodes represented in the network. An extend parameter can also be applied which will expand each anchor by the amount specified in both directions, offering flexibility for defining nodes. Regardless of the extend amount, nodes will be represented by the minimum start position and maximum end position of the anchors that define it.

Edge Creation

Edges are created by first initializing a tree-based map of node id keys, each referencing a list of interactions. As interactions maintain a reference to their corresponding anchors and anchors maintain a reference to the nodes they are assigned in the node creation step, this map is created by iterating over each interaction once. For each iteration, the key for the map is determined by concatenating the smallest integer node id with the largest node id (in that order) using a delimiter and the interaction is added to the list referenced by the key in the map. If both node ids are the same or one of anchors does not reference a node, then the interaction is not included. Once the map is created, edges are created by iterating over the keys and values in the map, using the key to determine the nodes to use for each edge. Finally, the edges

Algorithm 1 Node Creation with Interaction Anchors

```
anchors[];                                   //array of interaction anchors
nodelist = new List();
chrgroups[] = separateByChromosome(anchors);

for i = 0 to chrgroups.length do
    sortByStartPosition(chrgroups[i]);
end for

for i = 0 to chrgroups.length do
    sortedanchors = chrgroups[i];
    anchorlist = new List();
    maxend = sortedanchors[0].endPosition;
    anchorlist.add(sortedanchors[0]);
    for j = 1 to sortedanchors.length do
        if sortedanchors[j].startPosition ≤ maxend + extend * 2 then
            anchorlist.add(sortedanchors[j]);
        else
            nodelist.add(new Node(anchorlist));
            anchorlist = new List();
            anchorlist.add(sortedanchors[j]);
        end if
        maxend = max(maxend, sortedanchors[j].endPosition);
    end for
    nodelist.add(new Node(anchorlist));
end for
return nodelist;
```

are filtered based on filtering parameters provided, removing them from the final network.

Algorithm 2 Edge Creation

$interactions[]$; //array of interactions in the network
$edgemap$; //map of node id keys and interaction lists

for $i = 0$ to $interactions.length$ **do**
  $node1id = interactions[i].getAnchor1().getNode()$;
  $node2id = interactions[i].getAnchor2().getNode()$;
  if $node1id \neq node2id$ **then**
    $minid = min(node1id, node2id)$;
    $maxid = max(node1id, node2id)$;
    $key = minid + ",\," + maxid$;
    if $edgemap.containsKey(key)$ **then**
      $edgemap.put(key, newList())$
    end if
    $edgemap.get(key).add(interaction[i])$;
  end if
end for

for each key in $edgemap$ **do**
  $nodeids[] = split(K,",")$;
  $createEdge(nodeids[0], nodeids[1], edgemap.get(K))$;
end for

Connected Component Discovery

With the nodes and edges created, connected components are determined in linear time by maintaining a Boolean array of visited nodes and performing Breadth-First Search on every node that has not yet been visited. The algorithm for this process simply iterates over the list of nodes where in each iteration, if the node has not been visited yet, a breadth-first search is performed putting all nodes and edges visited into the same connected component. Nodes visited when performing a breadth-first search are marked as visited such that breadth-first search is not repeated on the same component. After all nodes have been visited, all connected components in the network

have been identified. Finally, single node components are removed from the network as they do not provide any interaction information and have proven to significantly increase the computational time for node annotation which is database query driven.

Algorithm 3 Connected Component Discovery

```
nodes[];                              //array of nodes in the network
visited[];                            //boolean array of visited nodes
componentlist;                        //list of connected components discovered

for i = 0 to nodes.length do
    if visited[nodes[i].id] = FALSE then
        component = breadthFirstSearch(nodes[i]);
        markNodesAsVisited(component, visited);
        componentlist.add(component);
    end if
end for
return componentlist;
```

Network Annotation

Annotations on the network are performed using database queries where the list of annotations are first converted to genomic coordinates and are then queried against the nodes in the network to determine the overlap between them, checking that chromosomes are equivalent and start positions of the annotation and node are less than or equal to the end positions of each other. Once the list of nodes is determined, an index is then saved in the database for future visualizations and analyses.

Target Discovery

Target discovery is performed by assigning source and target annotations and performing breadth first search on every node annotated with a source annotation. All shortest paths from nodes with the source annotations to nodes with target annotations are maintained and reported.

Annotation Interaction Enrichment

Annotation Interaction Enrichment considers the annotations among all edges within the component threshold and counts the number of times an edge has a certain pairing of annotations as observed counts. Expected counts can be determined by one of two methods of the user's choosing:

Theoretical: The theoretical expected number of edges with each configuration of annotations are determined using the following formula where $|a|$ represents the number of nodes with annotation a, $|b|$ represents the number of nodes with annotation b, $|E|$ represents the total number of edges in the network, and $|N|$ represents the total number of nodes in the network:

$$\mathrm{E}[a,b] = 2|E| \left[\frac{|a|}{|N|}\right] \left[\frac{|b|}{|N|-1}\right]$$

When the annotations are the same, the following formula is used instead:

$$\mathrm{E}[a,a] = |E| \left[\frac{|a|}{|N|}\right] \left[\frac{|a|-1}{|N|-1}\right]$$

Permutation: Expected frequencies derived from permutations are calculated by randomly reassigning nodes in the network with each annotation a number of times specified by the user (between 1 and 100,000). The frequency of interactions are calculated between annotations and the average over all permutations is used as the expected number of edges for each annotation pair. P-Values using the null distribution derived from the permutations are calculated by performing a two-tailed test, counting the number of permutations falling greater than or less than the absolute value of the observed frequency in the distribution.

For each method, a heatmap is produced by using the $\log_2$ ratio of the observed over expected values. P-Values using the binomial test are also calculated and provided.

Case Study: Network Construction

The MCF7 interaction network was constructed using replicate 4 of MCF7 ChIA-PET data from ENCODE[1], accession GSM970209. To define the nodes in the network, we used peaks from DNASE-Seq which define open chromatin sites. For this, two replicates of MCF7 DNASE-Seq bam files from ENCODE[1], accession GSM816627, were merged using SAMTools[1] [2] and peaks were called using MACS2[2] [3]. The interaction network was then constructed by extending the anchors by 250bp and using default values for the remaining parameters[3].

Case Study: Network Annotation

The MCF7 network was annotated with the 761 Non-Coding Variants (NCVs) for MCF7 obtained from COSMIC[4] [4] (cancer.sanger.ac.uk) as well as with the following gene lists:

Known Oncogenes: Known oncogenes were selected as the union of (1) Genes tagged by 'Entrez Query: Oncogene' in CancerGenes [5], manually reassigning genes which also matched 'Entrez Query: Tumour Suppressor'. (2) genes amplified and overexpressed in cancer from [6], (3) essential genes from [7].

Known Tumor Suppressor Genes: Known tumor suppressor genes were selected as the union of (1) known recessive tumor suppressor genes according to the Cancer Gene Census [8], (2) homozygously inactivated genes observed by whole genome sequencing in COSMIC [4], (3) genes tagged by 'Entrez Query: Tumour Suppressor' in CancerGenes [5], manually reassigning genes matching 'Entreze Query: Oncogene', (4) Human protein coding TSGs from TSGene database [9].

---

[1]SAMtools Version: 1.2 (Using htslib 1.2.1)

[2] MACS2 (2.1.0.20150731) **Parameters:** -g 'hs' –nomodel –shift -100 –extsize 200 -B –broad –keep-dup=1

[3]**Node/Anchor Extension:** 250, **Min Paired Ends/Score Per Edge:** 0, **Interactions:** Intra-Chromosome, **Max Intrachromosome Distance (bp):** 1000000

[4]GRCh 37 Archive of the Cell Lines Project: `http://grch37-cancer.sanger.ac.uk/cell_lines/sample/overview?id=905946`

Breast Cancer Genes Associated with Good & Poor Prognosis: Genes selected to be associated with good and poor prognosis of breast cancer were selected based on those described by [10].

Known Oncogenes & Tumor Suppressor Genes Identified by Davoli: The top 300 tumor suppressor genes (q value < 0.18) and top 250 oncogenes (q value < 0.22) were selected from the genes identified by Davoli et al. [11].

Case Study: Promoter Association Methods

Below we define the three methods used for selecting promoters.

Nearest TSS: Promoters defined by nearest transcription start sites (TSS) were defined with mutations found in the network (for comparison purposes) and selecting the nearest TSS of genes identified by UCSC's RefSeq annotation database [12].

Direct Target: Promoters identified by direct targets were selected by first performing Target Discovery to obtain the shortest paths from the NCV to promoters (2kb upstream/downstream from the TSS) using ChIA-PET interactions. Direct targets were then selected based on paths with 0 to 1 edges between the node harboring the NCV and the node overlapping the target promoter.

Indirect Target: Indirect targets were identified using the same target discovery used for direct targets but using different cutoffs for the edges found between the NCV and target promoter. Performing an enrichment analysis using fisher's exact test with the cancer gene list over all possible cutoffs from 2 to 16 (after removing redundancies) revealed promoters 2 to 4 edges away from the NCV in the network maximized the enrichment significance (S3 Fig.) and genes were therefore selected based on this criteria.

Case Study: Expression Comparisons Between Nearest Genes and Network-Related Genes

To estimate the magnitude and significance of cancer-related differential expression of the genes identified as potential targets of COSMIC somatic mu-

tations, we performed a negative binomial regression/ANOVA on expression data obtained form The Cancer Genome Atlas (TCGA) database using the R/MASS::glm.nb procedure. Specifically, we tested for significant differences between samples coded as tumor (N=901) and normal (N=92) using base-2 logs of normalized counts of transcripts of these genes. We then graphically compared the resulting regression coefficients, which estimate fold changes of expression, between (1) genes located physically nearest to each mutation, (2) genes whose promoter directly overlaps a mutation or is directly connected to a network node overlapping a mutation (i.e., direct interaction), and (3) genes whose promoter is connected to a network node separated by 2 to 4 links (i.e., indirect interaction), after filtering out instances where the nearest TSS was also part of the network, and making sure no gene was duplicated in either of these three categories. Finally, we repeated the above procedure comparing expression data between MCF7 and MCF10A bulk mRNA-seq samples [13] obtained from the Gene Expression Omnibus, accession number GSE52712, using the online tool GEO2R to compute regression coefficients and P-values.

Case Study: Enrichment of Cancer Related Genes

To determine whether a set of genes is enriching for a particular set of cancer related genes (e.g. Tumor Suppressor or Oncogenes) we used Fisher's exact test by providing the 2x2 contingency table of values counting the number of genes selected (e.g. genes selected by direct or indirect targets), the number of these genes in the intersection of the cancer related gene set of interest among the universe of all genes which was calculated to be the intersection of genes in the UCSC refseq database and the genes identified in TCGA. S2 Table provides the number of genes in each of these categories to calculate the 2x2 contingency table used for the enrichment of non-coding variants and cancer related gene sets. For calculating the P-Value, we used the fisher.test method in R under the two-tailed alternative hypothesis.

Case Study: Gene Co-Expression Analysis

To compare the co-expression of genes between direct, indirect, and genes without any chromatin interactions between them, we used expression data from TCGA tumor samples and filtered all genes with too many missing values or zero variance. After filtering, we calculated the matrix of pearson

correlations as well as a matrix representing the minimum number of edges between every pair genes found within the network. The correlations were then categorized as “Direct”, “Indirect (2-4)”, “All Indirect”, and ”No Interaction” based on having minimum edges of 1, $2-4$, >1, and having no path of edges respectively within the corresponding minimum edge matrix.

References

[1] Consortium EP. An integrated encyclopedia of DNA elements in the human genome. Nature. 2012;489(7414):57-74. doi: 10.1038/nature11247. PubMed PMID: 22955616; PubMed Central PMCID: PMC3439153.

[2] Li H, Handsaker B, Wysoker A, Fennell T, Ruan J, Homer N, et al. The Sequence Alignment/Map format and SAMtools. Bioinformatics. 2009;25(16):2078-9. doi: 10.1093/bioinformatics/btp352. PubMed PMID: 19505943; PubMed Central PMCID: PMC2723002.

[3] Zhang Y, Liu T, Meyer CA, Eeckhoute J, Johnson DS, Bernstein BE, et al. Model-based analysis of ChIP-Seq (MACS). Genome Biol. 2008;9(9):R137. doi: 10.1186/gb-2008-9-9-r137. PubMed PMID: 18798982; PubMed Central PMCID: PMC2592715.

[4] Forbes SA, Beare D, Gunasekaran P, Leung K, Bindal N, Boutselakis H, et al. COSMIC: exploring the world’s knowledge of somatic mutations in human cancer. Nucleic acids research. 2015;43(Database issue):D805-11. doi: 10.1093/nar/gku1075. PubMed PMID: 25355519; PubMed Central PMCID: PMC4383913.

[5] Higgins ME, Claremont M, Major JE, Sander C, Lash AE. CancerGenes: a gene selection resource for cancer genome projects. Nucleic acids research. 2007;35(Database issue):D721-6. doi: 10.1093/nar/gkl811. PubMed PMID: 17088289; PubMed Central PMCID: PMC1781153.

[6] Santarius T, Shipley J, Brewer D, Stratton MR, Cooper CS. A census of amplified and overexpressed human cancer genes. Nature reviews Cancer. 2010;10(1):59-64. doi: 10.1038/nrc2771. PubMed PMID: 20029424.

[7] Solimini NL, Xu Q, Mermel CH, Liang AC, Schlabach MR, Luo J, et al. Recurrent hemizygous deletions in cancers may optimize proliferative potential. Science. 2012;337(6090):104-9.

[8] Futreal PA, Coin L, Marshall M, Down T, Hubbard T, Wooster R, et al. A census of human cancer genes. Nat Rev Cancer. 2004;4(3):177-83. doi: 10.1038/nrc1299. PubMed PMID: 14993899; PubMed Central PMCID: PMC2665285.

[9] Zhao M, Sun J, Zhao Z. TSGene: a web resource for tumor suppressor genes. Nucleic acids research. 2013;41(Database issue):D970-6. doi: 10.1093/nar/gks937. PubMed PMID: 23066107; PubMed Central PMCID: PMC3531050.

[10] Inaki K, Menghi F, Woo XY, Wagner JP, Jacques PE, Lee YF, et al. Systems consequences of amplicon formation in human breast cancer. Genome research. 2014;24(10):1559-71. doi: 10.1101/gr.164871.113. PubMed PMID: 25186909; PubMed Central PMCID: PMC4199368.

[11] Davoli T, Xu AW, Mengwasser KE, Sack LM, Yoon JC, Park PJ, et al. Cumulative haploinsufficiency and triplosensitivity drive aneuploidy patterns and shape the cancer genome. Cell. 2013;155(4):948-62. doi: 10.1016/j.cell.2013.10.011. PubMed PMID: 24183448; PubMed Central PMCID: PMC3891052.

[12] Rosenbloom KR, Armstrong J, Barber GP, Casper J, Clawson H, Diekhans M, et al. The UCSC Genome Browser database: 2015 update. Nucleic acids research. 2015;43(Database issue):D670-81. doi: 10.1093/nar/gku1177. PubMed PMID: 25428374; PubMed Central PMCID: PMC4383971.

[13] Rothwell DG, Li Y, Ayub M, Tate C, Newton G, Hey Y, et al. Evaluation and validation of a robust single cell RNA-amplification protocol through transcriptional profiling of enriched lung cancer initiating cells. BMC Genomics. 2014;15:1129. doi: 10.1186/1471-2164-15-1129. PubMed PMID: 25519510; PubMed Central PMCID: PMC4320548.
